# Germinated Foxtail Millet Protein Attenuates CCl_4_-Induced Acute Liver Injury with Reduced Hepatic Oxidative Stress and Gut Microbiota Remodeling

**DOI:** 10.3390/foods15213784

**Published:** 2026-10-22

**Authors:** Jingjing Li, Yunpeng Shen, Fangfang Min, Xiao Chen, Hongge Yu, Tian Yu, Zicheng Yin, Sha Huang

**Affiliations:** 1 State Key Laboratory of Food Science and Resources, Nanchang University, Nanchang 330047, China; lijingjing@email.ncu.edu.cn (J.L.); syp@email.ncu.edu.cn (Y.S.); min2fang@email.ncu.edu.cn (F.M.); chenxiao6141@email.ncu.edu.cn (X.C.); yhg@email.ncu.edu.cn (H.Y.); yutian@email.ncu.edu.cn (T.Y.); 17368105006@163.com (Z.Y.); 2 College of Food Science and Technology, Nanchang University, Nanchang 330031, China; 3 Sino-German Joint Research Institute, Nanchang University, Nanchang 330047, China; 4 Lishui Institute of Agriculture and Forestry Sciences, Lishui 323000, China

**Keywords:** germinated foxtail millet protein, carbon tetrachloride, acute liver injury, oxidative stress, gut microbiota

## Abstract

Foxtail millet (*Setaria italica*) is a nutrient-rich cereal whose bioactive properties can be enhanced by germination. However, whether germinated foxtail millet protein (GR) pretreatment protects against carbon tetrachloride (CCl_4_)-induced acute liver injury and how this response relates to the gut microbiota remain unclear. Mice received oral GR at 0.5 or 1 g/kg for 14 days before CCl_4_ challenge. GR reduced the liver index and serum aspartate aminotransferase and alkaline phosphatase activities, alleviated hepatocellular necrosis and inflammatory-cell infiltration, increased hepatic superoxide dismutase activity, and decreased malondialdehyde content. Transcriptomic analysis of the high-dose GR (HGR) group identified 80 differentially expressed genes relative to the Model group, with enrichment in the cytokine-cytokine receptor interaction, TGF-β signaling, chemokine signaling, and amino acid metabolism. RT-qPCR confirmed higher *Cx3cr1* and lower *Got1* transcript abundance with HGR. Moreover, 16S rRNA sequencing revealed overall differences in cecal microbial community composition among the Control, Model, and HGR groups. Correlation analysis further linked liver injury and oxidative stress indicators with HGR-associated bacterial genera and hepatic genes. Collectively, GR pretreatment attenuated CCl_4_-induced acute liver injury and improved hepatic oxidative status. while the HGR-associated transcriptional and microbial changes provide candidate targets for further mechanistic investigation. These findings support further evaluation of GR for potential food applications.

## 1. Introduction

Foxtail millet (*Setaria italica*) is a nutrient-rich cereal containing proteins, dietary fiber, unsaturated fatty acids, polysaccharides, and polyphenols with antioxidant, anti-inflammatory, and metabolic regulatory potential [1]. Germination activates endogenous enzymes and can increase γ-aminobutyric acid (GABA), phenolic compounds, flavonoids, and antioxidant activity while reducing antinutritional factors [2,3]. Germinated or fermented-germinated foxtail millet whole-grain flour has also been reported to improve intestinal barrier function and alleviate gut microbiota dysbiosis in mice [4]. However, whole-grain flour contains multiple bioactive components, making it difficult to determine the specific contribution of its protein fraction.

Foxtail millet protein is a potential source of antioxidant and anti-inflammatory peptides. Peptides released during simulated gastrointestinal digestion of germinated and heat-treated foxtail millet proteins reduced reactive oxygen species production, enhanced cellular antioxidant defenses, and inhibited inflammatory mediators in cell models [5]. Recent peptidomic analysis further confirmed the antioxidant potential of foxtail millet bran protein hydrolysates [6]. Millet bran protein hydrolysate alleviated hepatic steatosis by regulating fatty acid uptake [7], whereas dietary millet bran protein improved intestinal barrier function and metabolic disorders by remodeling the gut microbiota [8]. In addition, foxtail millet polyphenols ameliorated fatty liver disease in mice through changes in the intestinal microbiome [9]. These findings indicate that millet-derived proteins and other bioactive components can influence hepatic metabolism and intestinal homeostasis. Nevertheless, the hepatoprotective effects of germinated foxtail millet protein and its potential gut–liver mechanisms in toxicant-induced liver injury remain unclear.

Carbon tetrachloride (CCl_4_) is commonly used to induce experimental liver injury. Its hepatic metabolism generates reactive radicals that cause lipid peroxidation, oxidative stress, inflammation, hepatocyte necrosis, and fibrosis [10]. Gut microbiota dysbiosis and intestinal barrier disruption can aggravate liver injury by increasing the portal translocation of lipopolysaccharide and activating hepatic inflammatory signaling. Conversely, microbial metabolites such as short-chain fatty acids and bile acids can regulate intestinal barrier integrity, hepatic metabolism, and immune responses [11,12]. Recent CCl_4_ studies have demonstrated associations among differential bacterial taxa, hepatic transcriptomic or metabolic alterations, and liver injury indicators [13,14,15,16]. These findings suggest that the gut microbiota may be involved in the response to dietary interventions during CCl_4_-induced liver injury.

Therefore, this study evaluated the protective effects of germinated foxtail millet protein (GR) pretreatment against CCl_4_-induced liver injury in mice. Liver transcriptomic and gut microbiota analyses were used to identify HGR-associated gene-expression and microbial changes accompanying the biochemical phenotype. Correlations were examined to generate hypotheses for subsequent mechanistic investigation.

## 2. Materials and Methods

### 2.1. Materials

GR was the freeze-dried protein isolated obtained from germinated foxtail millet by alkaline extraction and acid precipitation. The same preparation batch as in our previous study [5] was used, and no enzymatic hydrolysis was performed before administration. The protein content of GR was 80.93% (*w*/*w*), as estimated using the bicinchoninic acid (BCA) assay. The detailed germination and protein isolation procedure is provided in the Supporting Information. CCl_4_ (98% purity), olive oil, and carboxymethylcellulose sodium (CMC-Na) were purchased from Shanghai Macklin Biochemical Technology Co., Ltd. (Shanghai, China). 4% paraformaldehyde was purchased from Biosharp Biotechnology Co., Ltd. (Hefei, China). Commercial kits for aspartate aminotransferase (AST), alkaline phosphatase (ALP), superoxide dismutase (SOD), and malonaldehyde (MDA) were purchased from Nanjing Jiancheng Bioengineering Institute (Nanjing, China). The bicinchoninic acid protein assay kit was purchased from Beyotime Biotech. Inc. (Shanghai, China).

### 2.2. Animals

Thirty-six specific pathogen-free (SPF) male BALB/c mice (6–8 weeks old) were purchased from Zhejiang Charles River Laboratory Animal Technology Co., Ltd. (Ningbo, China) (Certificate SCXK (Zhe) 2019-0001). All experiments were approved by the Ethics Committee of Jiangxi Institute of Materia Medica (Approval number: JXIMM 21-2207-1). Mice were housed in a controlled environment (20–26 °C, humidity 40–70%) with a regular 12 h dark/light cycle and free access to water.

### 2.3. Experimental Protocol

**Figure 1:**
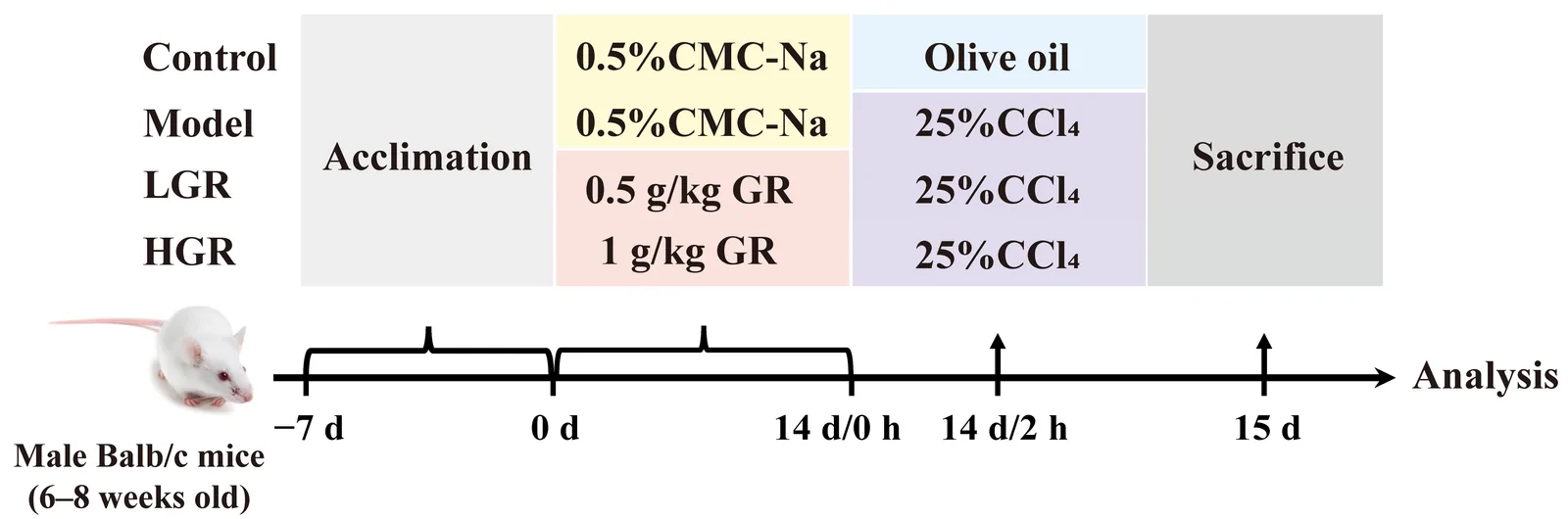
Design of the animal study on the hepatoprotective effects of germinated foxtail millet proteins (GR) pretreatment.

The CCl_4_-induced acute liver injury model was established with minor modifications from previous studies [14,17]. GR doses were selected with reference to reported in vivo studies of orally administered foxtail millet protein preparations [18,19,20]. The experimental design is shown in Figure 1. After one week of acclimation, the 36 experimental mice were individually numbered. The identification numbers were randomly ordered using Microsoft Excel, and the mice were allocated according to this order to four groups, with nine mice per group: Control, Model, low-dose GR (LGR), and high-dose GR (HGR). The individual mouse was considered the experimental unit. The LGR and HGR groups received GR by oral gavage at 0.5 and 1.0 g/kg body weight, respectively, once daily for 14 consecutive days. The control and model groups received an equivalent volume of 0.5% CMC-Na. Body weight was monitored during the experiment. Two hours after the final administration, mice in the model, LGR, and HGR groups received a single intraperitoneal injection of 25% (*v*/*v*) CCl_4_ in olive oil at 2 mL/kg body weight. Control mice received an equivalent volume of olive oil. Following injection, the mice were fasted for 24 h before scheduled terminal sampling. During this period, mice were checked every 3 h. The predefined humane endpoint criteria throughout the experiment were rapid loss of 15–20% of baseline body weight; absence of a response to painful stimuli; complete inability to stand or right themselves unaided; or abnormal respiration accompanied by a rectal temperature <35 °C. During the 24-h post-injection period, two mice in the Model group and two mice in the LGR group reached the predefined humane endpoints and were euthanized before scheduled terminal sampling. No necropsy or other investigation was performed to determine the underlying causes of their clinical deterioration. At the end of the 24-h period, the 32 surviving mice were weighed and euthanized: Control, *n* = 9; Model, *n* = 7; LGR, *n* = 7; and HGR, *n* = 9. Blood, liver, spleen, and cecal contents were collected from these animals. The liver and spleen were rinsed with ice-cold saline and weighed. Organ indices were calculated as follows: Organ index (%) = organ weight (g)/body weight (g) × 100%. Endpoint-specific sample availability is summarized in Table S1.

GR doses were expressed as the total mass of the freeze-dried protein isolate per kilogram of body weight. Human equivalent doses (HEDs) were estimated by body surface area normalization using the equation HED (mg kg^−1^ day^−1^) = mouse dose (mg kg^−1^ day^−1^) × 3/37, where 3 and 37 are the standard Km factors for mice and adult humans, respectively [21]. The GR doses of 0.5 and 1 g kg^−1^ day^−1^ corresponded to estimated HEDs of 40.5 and 81.1 mg kg^−1^ day^−1^, equivalent to approximately 2.43 and 4.86 g/day of the freeze-dried GR preparation for a 60-kg adult. For nutritional context, the EFSA population reference intake for protein in adults is 0.83 g kg^−1^ day^−1^, corresponding to 49.8 g/day for a 60-kg adult [22]. The HED estimates are provided for interspecies dose comparison, and the EFSA value is cited only as a nutritional reference; neither establishes a recommended human intake level for GR.

### 2.4. Histological Examination

The liver tissue was immersed in a 4% paraformaldehyde solution, subsequently embedded in paraffin, and subjected to hematoxylin-eosin (H&E) staining. H&E-stained liver sections from three mice per group were examined. For each mouse, three distinct microscopic fields were imaged at both 100× and 200× magnification utilizing an optical microscope (Nikon Corporation, Tokyo, Japan). Images at the two magnifications represented the same tissue regions. Histological assessment was descriptive and unblinded, focusing on hepatic architecture, hepatocellular necrosis, and inflammatory-cell accumulation. No semi-quantitative injury scoring or quantitative image analysis was performed.

### 2.5. Biochemical Analysis of Liver Tissues and Serum

All biochemical analyses were performed using samples collected only from animals that survived to scheduled terminal sampling. The four mice euthanized earlier upon reaching humane endpoints were not included in these analyses. After resting the blood at room temperature for 30 min, centrifugation was conducted at 4 °C, 3500 rpm for 15 min. The serum enzyme activities of AST and ALP were measured using commercially available kits. The 10% liver tissue homogenates were prepared by adding normal saline. Following centrifugation at 4 °C, 2500× *g* for 10 min, protein concentrations in the supernatants were determined using the bicinchoninic acid protein assay kit. Subsequently, the supernatants were assessed for SOD activity and MDA content using commercially available kits, following the manufacturer’s protocol.

### 2.6. RNA-Seq and RT-qPCR Analysis

Total RNA was extracted from liver samples of four randomly selected mice in each of the Control, Model, and HGR groups (*n* = 4 biological replicates per group) using TRIzol reagent (Sigma-Aldrich, MO, USA). Sequencing libraries were prepared through RNA purification and fragmentation, reverse transcription, and PCR amplification, followed by library quality control and paired-end sequencing on an Illumina NovaSeq system (Illumina, San Diego, CA, USA). Adapter sequences were removed using Cutadapt, with a minimum overlap of 10 bp and a maximum mismatch rate of 20%. Reads with an average Phred quality score below 20 were discarded. After filtering, 33.71–46.39 million clean reads, corresponding to 5.09–7.00 Gb of sequence data, were retained per sample. Clean reads were aligned to the mouse reference genome GRCm39 (Mus_musculus.GRCm39.dna.toplevel.fa) using HISAT2, with total mapping rates of 94.82–96.88%. Sample-level sequencing and quality-control statistics are provided in Supplementary Table S2. Gene level read counts were obtained using HTSeq, and FPKM values were calculated to describe gene expression levels. Sample-to-sample expression similarity was assessed using Pearson correlation analysis and principal component analysis. Differential gene expression was analyzed using DESeq2. Gene level *p* values were adjusted for multiple testing using the Benjamini–Hochberg procedure. Genes with an adjusted *p* value < 0.05 and |log_2_ fold change| > 1 were defined as differentially expressed. KEGG enrichment analysis was performed using the resulting DEG set. The transcriptome sequencing service of liver tissues from mice was provided by Per-sonalbio Technology Co., Ltd., (Shanghai, China) and was analyzed using the free online platform Personalbio GenesCloud (https://www.genescloud.cn). The original FASTQ in-formation for the samples was uploaded to the NCBI repository (Sample accession number: PRJNA1163738). For RT-qPCR analysis of *Cx3cr1* and *Got1* (*n* = 4 mice per group in Control, Model, and HGR), extracted RNA was reverse-transcribed into cDNA using a reverse transcription kit according to the manufacturer’s instructions. qPCR was performed using TB Green Premix Ex Taq II (Tli RNaseH Plus, Takara Biomedical Technology (Beijing) Co., Ltd., Beijing, China) on a QuantStudio 5 real-time PCR system. The primers used in this study are listed in Table S3. Relative gene expression levels were normalized to β-actin and calculated using the 2^−ΔΔCt^ method.

### 2.7. 16S rRNA Sequencing

Genomic DNA was extracted from the cecal contents of six randomly selected mice in each of the Control, Model, and HGR groups (*n* = 6 per group), and its purity and concentration were evaluated. Subsequently, the extracted genomic DNA was amplified using barcoded conventional primer pairs 338F (5′-ACTCCTACGGGAGGCAGCAG-3′) and 806R (5′-GGACTACHVGGGTWTCTAAT-3′) targeting the bacterial 16S rRNA gene V3-V4 hypervariable region. The resulting PCR products were then cleaned, quantified, and homogenized to create a sequencing library. Sequencing was performed on the Illumina MiSeq platform (Shanghai Majorbio BioPharm Technology Co., Ltd., Shanghai, China). Raw sequences underwent quality control using fastp software (v0.19.6) and FLASH software (v1.2.11) for splicing. Subsequently, UPARSE software (v11) was used to cluster the sequences into operational taxonomic units (OTUs) and remove chimeras based on 97% similarity. PCA was performed on Z-score-standardized OTU abundance data, and PCoA was based on Bray–Curtis dissimilarities. Overall differences among the Control, Model, and HGR groups were assessed by analysis of similarities (ANOSIM), using Euclidean distances for the analysis accompanying PCA and Bray–Curtis dissimilarities for the analysis accompanying PCoA. Each ANOSIM test used 999 permutations. Overall differences in genus level relative abundances among the Control, Model, and HGR groups were assessed using the Kruskal–Wallis test, followed by FDR correction across the tested genera. FDR-adjusted *p* < 0.05 was considered statistically significant. Linear discriminant analysis effect size (LEfSe) was performed on normalized taxonomic abundance data from the phylum to genus levels using the all-against-all multiclass strategy, with an unadjusted *p*-value threshold of 0.05 and a logarithmic LDA score threshold of 2.0. No additional FDR correction was applied to the LEfSe analyses. The resulting data was analyzed using the online platform available at http://cloud.majorbio.com/. The original FASTQ information for the samples was uploaded to the NCBI repository (Sample accession number: PRJNA1163529).

### 2.8. Statistical Analysis

Data were statistically analyzed and graphed using Graphpad Prism 9, Origin 2021, Personalbio GenesCloud, and Majorbio Cloud. Liver index, spleen index, serum AST and ALP, and hepatic SOD and MDA, and relative *Cx3cr1* and *Got1* expression measured by RT-qPCR were analyzed using one-way ANOVA followed by Tukey’s multiple-comparisons test. Between-group mean differences, Tukey-adjusted 95% confidence intervals, and adjusted *p* values are reported in Table S4. Body-weight measurements were summarized descriptively at each recorded time point. A correlation analysis was performed using Spearman analysis, and further detailed is provided in the Supporting Information. Data are presented as means ± (SD). *p* < 0.05 was regarded as statistically significant.

## 3. Results

### 3.1. GR Pretreatment Attenuated CCl_4_-Induced Liver Injury

**Figure 2:**
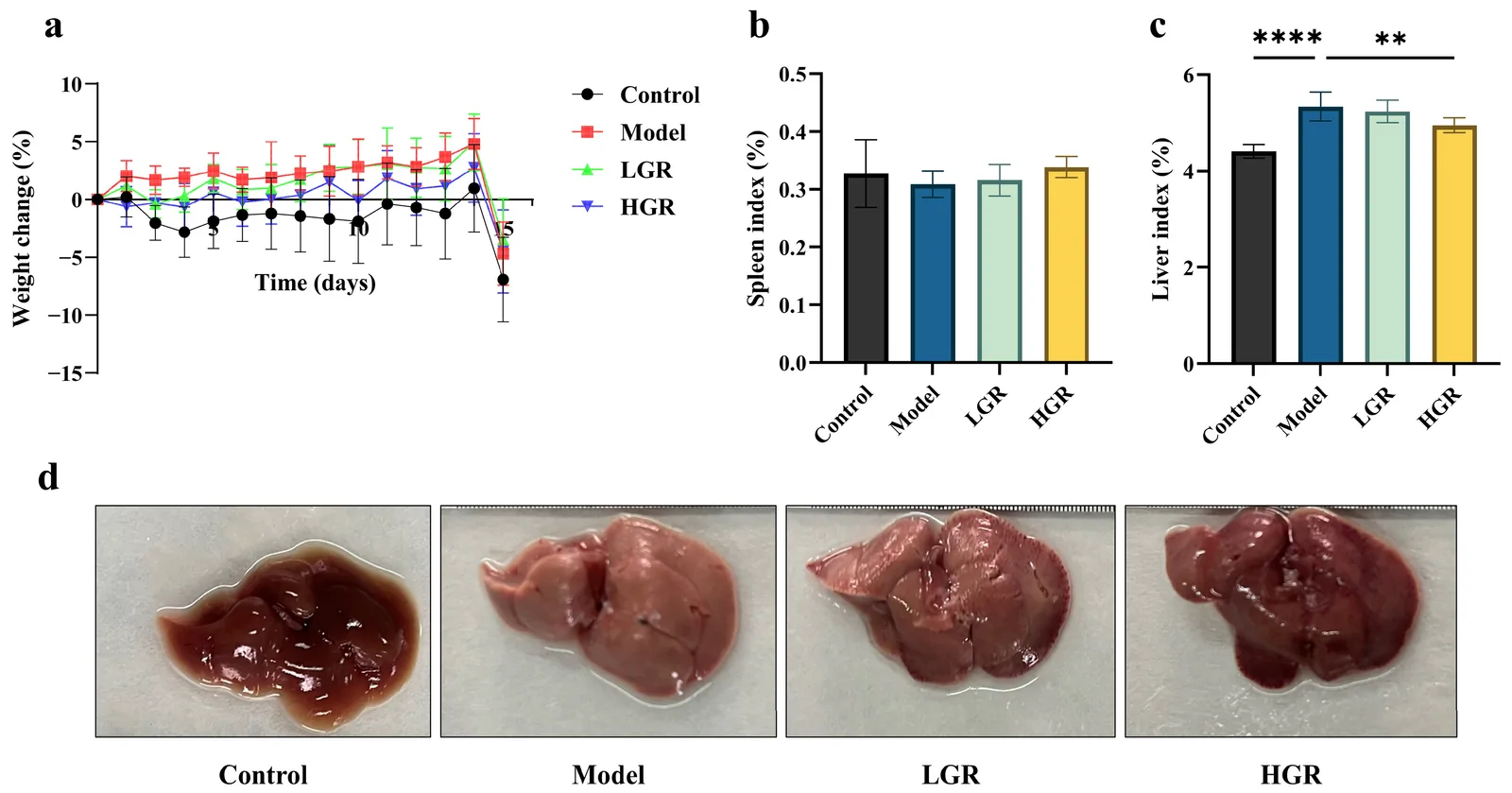
GR pretreatment alleviated CCl_4_-induced liver hepatomegaly. (**a**) Changes in body weight of mice; (**b**) Spleen index calculated in groups; (**c**) Liver index calculated in groups; (**d**) Representative liver photograph. Data were expressed as mean ± SD, with Control, *n* = 9; Model, *n* = 7; LGR, *n* = 7; and HGR, *n* = 9. ** *p* < 0.01, **** *p* < 0.0001.

**Figure 3:**
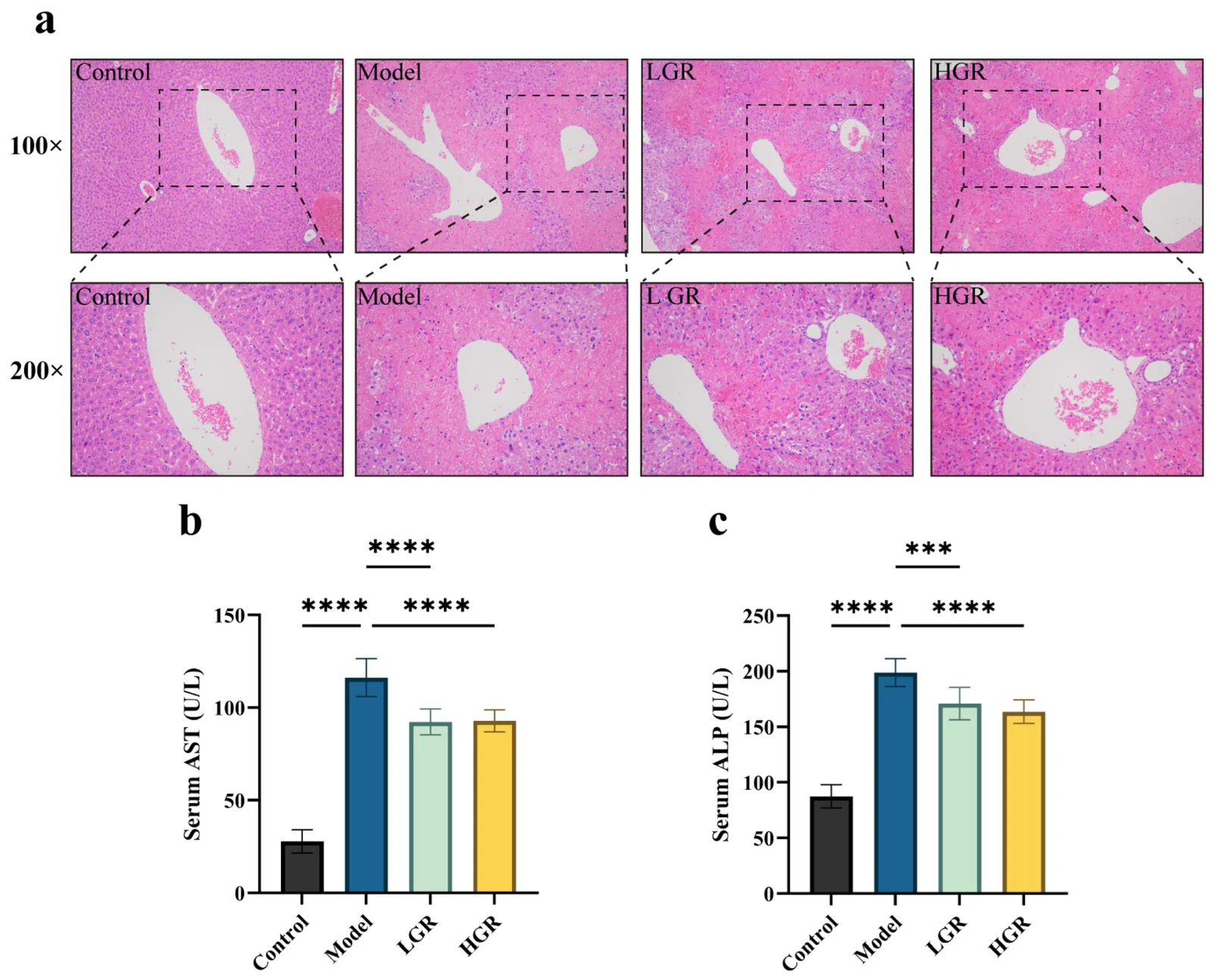
GR pretreatment ameliorated CCl_4_-induced changes in histopathological and serum injury-associated enzyme activities. (**a**) Representative H&E-stained liver sections. Sections from three mice per group were examined, with three distinct fields per mouse imaged at both 100× and 200× magnification. Histological findings were assessed qualitatively without scoring or quantitative image analysis; (**b**) serum AST levels measured in the groups; (**c**) serum ALP levels measured in the groups. Data were expressed as mean ± SD, with Control, *n* = 9; Model, *n* = 7; LGR, *n* = 7; and HGR, *n* = 9. *** *p* < 0.001, **** *p* < 0.0001.

Body weight was monitored throughout the experiment. During the 14-day pretreatment period before CCl_4_ exposure, neither GR-treated group showed sustained body weight loss (Figure 2a). Spleen indices measured at terminal sampling were similar across groups (Figure 2b). CCl_4_ exposure increased the liver index in the Model group compared with the Control group, whereas HGR pretreatment significantly attenuated this increase (Figure 2c). Representative gross liver images are shown in Figure 2d. Representative H&E images showed preserved hepatic architecture in the Control group, whereas the Model group exhibited pericentral hepatocellular necrosis and inflammatory-cell accumulation. Images from the GR-pretreated groups appeared to show less extensive necrosis and less prominent inflammatory-cell accumulation than those from the Model group (Figure 3a). Serum AST and ALP activities were elevated in the Model group compared with the Control group. GR pretreatment significantly reduced these enzyme activities compared with the Model group (Figure 3b,c).

### 3.2. HGR Pretreatment Altered Hepatic Gene Expression and Improved Hepatic Oxidative Status

**Figure 4:**
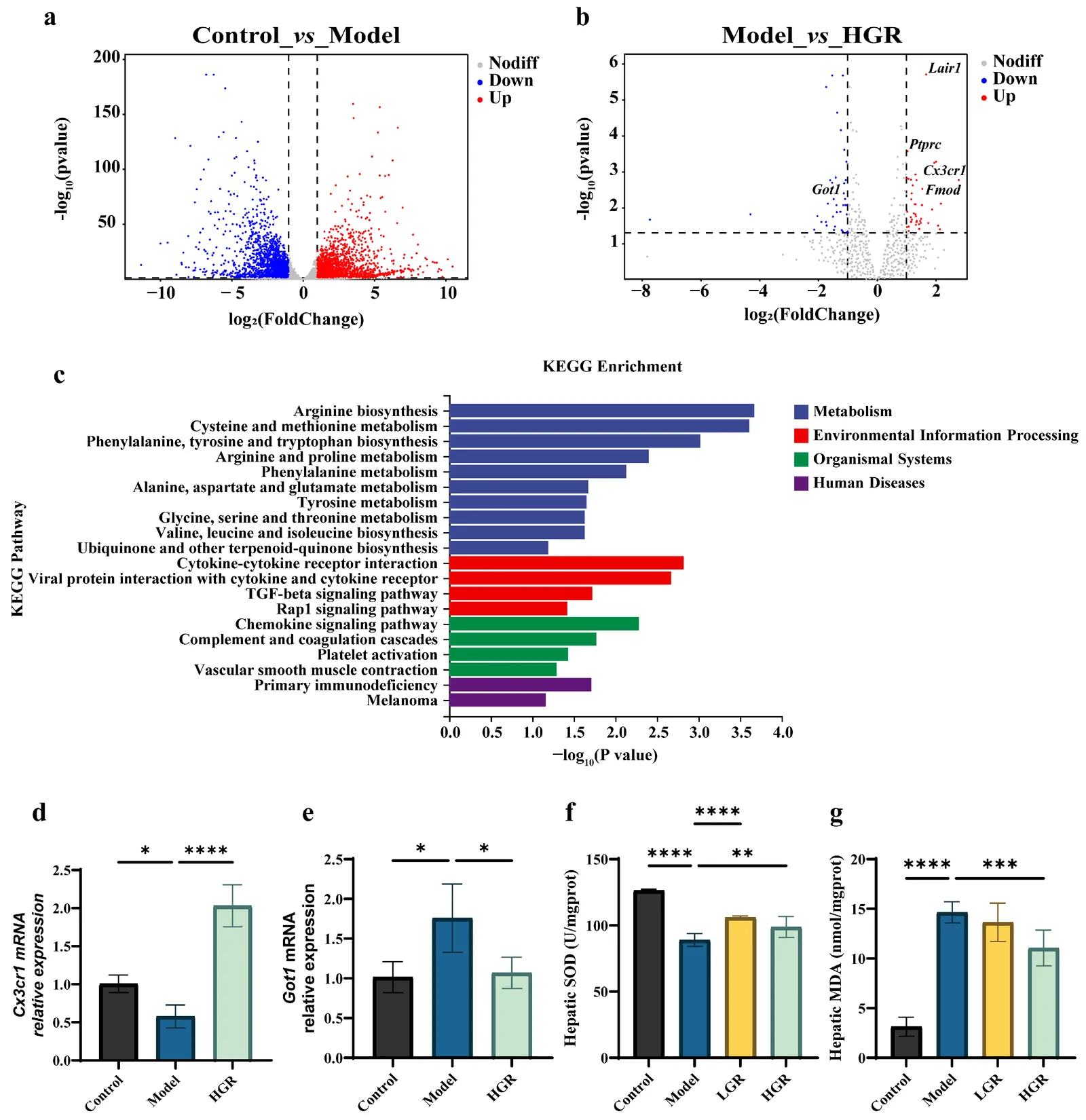
HGR pretreatment altered hepatic gene expression and improved hepatic oxidative status. (**a**) Volcano plot of hepatic gene expression in the Model group compared with the Control group; (**b**) volcano plot of hepatic gene expression in the HGR group compared with the Model group; The vertical dashed lines in (**a**) and (**b**) indicate log_2_ fold change thresholds of −1 and +1, while the horizontal dashed lines indicate an adjusted *p* value threshold of 0.05; (**c**) Exploratory KEGG enrichment analysis of DEGs between the HGR and Model groups; (**d**) relative hepatic mRNA expression of Cx3cr1 determined by RT-qPCR; (**e**) relative hepatic mRNA expression of Got1 determined by RT-qPCR; (**f**) hepatic SOD activity; (**g**) hepatic MDA content. RNA-seq and RT-qPCR analyses (**a**–**e**) included four mice in each of the Control, Model, and HGR groups (*n* = 4 per group). Data in (**f**,**g**) are presented as mean ± SD, with Control, *n* = 9; Model, *n* = 7; LGR, *n* = 7; and HGR, *n* = 9. * *p* < 0.05, ** *p* < 0.01, *** *p* < 0.001, **** *p* < 0.0001.

To characterize hepatic transcriptional responses to HGR pretreatment, RNA sequencing (RNA-seq) was performed in the Control, Model, and HGR groups. Transcriptome analysis identified 4285 differentially expressed genes (DEGs) in the Model group compared with the Control group (2249 upregulated and 2036 downregulated) and 80 DEGs in the HGR group compared with the Model group (42 upregulated and 38 downregulated) (Figure 4a,b). Among these DEGs, *Cx3cr1*, *Lair1*, *Fmod*, and *Ptprc* were upregulated, whereas *Got1* was downregulated in the HGR group compared with the Model group. Exploratory KEGG enrichment analysis highlighted cytokine–cytokine receptor interaction, TGF-β signaling, chemokine signaling, and amino acid metabolic pathways, including cysteine and methionine metabolism (Figure 4c). RT-qPCR analysis showed higher hepatic *Cx3cr1* mRNA expression and lower *Got1* mRNA expression in the HGR group compared with the Model group, consistent with the RNA-seq findings (Figure 4d,e). Both LGR and HGR increased hepatic SOD activity compared with the Model group. HGR reduced hepatic MDA content, whereas the LGR–Model comparison was not statistically significant (Figure 4f,g; Table S4). These biochemical observations indicate im-proved antioxidant defense with both doses and reduced lipid peroxidation with HGR.

### 3.3. HGR Altered the Cecal Microbial Community in Mice with Acute Liver Injury

**Figure 5:**
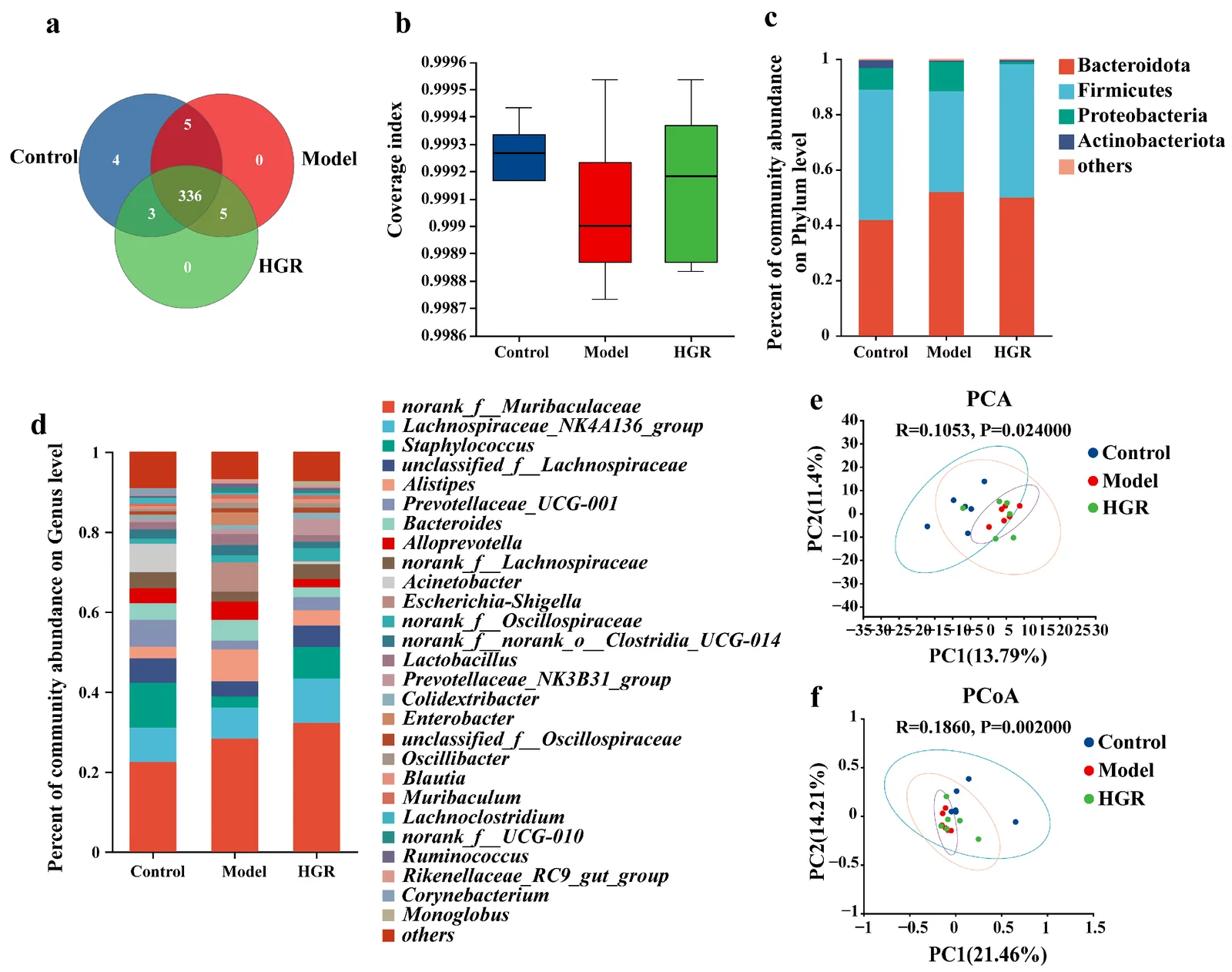
HGR pretreatment altered gut microbiota composition in mice with CCl_4_-induced liver injury. (**a**) Venn diagram showing shared and unique operational taxonomic units (OTUs); (**b**) Sequencing coverage index; (**c**) Relative bacterial abundance at the phylum level; (**d**) Relative bacterial abundance at the genus level; (**e**) Principal component analysis (PCA) based on OTU-level profiles; (**f**) Principal coordinates analysis (PCoA) based on OTU-level profiles. PCA was performed on Z-score-standardized OTU abundance data, and PCoA was based on Bray–Curtis dissimilarities. The displayed R statistics and *p*-values were obtained from ANOSIM using Euclidean and Bray–Curtis distances, respectively, with 999 permutations per test. Analyses included six mice in each of the Control, Model, and HGR groups (*n* = 6 per group).

16S rRNA gene sequencing characterized cecal microbial composition in the Control, Model, and HGR groups. The number of valid sequences for each sample was 29,994 after sampling, and 353 OTUs were obtained after clustering the sequences of all the samples (Figure 5a). The Coverage index was greater than 99.8% (Figure 5b), indicating that the amount of sequencing data was sufficient, and the detection ratio of mouse gut microbial community was close to saturation, which could cover most of the species in the samples. The four predominant phyla, Bacteroidota, Firmicutes, Proteobacteria, and Actinobacteriota, were abundant and shared by all groups. (Figure 5c). At the genus level, the order of the first nine genera was consistent across all groups (Figure 5d). PCA and PCoA visualized variation in cecal microbial community composition among the Control, Model, and HGR groups. ANOSIM detected significant overall differences among the three groups using both Euclidean distances (R = 0.1053, *p* = 0.024) and Bray–Curtis dissimilarities (R = 0.1860, *p* = 0.002) (Figure 5e,f). 

### 3.4. Differential Bacteria Genera and Exploratory Correlation with Biochemical Indicators and Hepatic Genes

**Figure 6:**
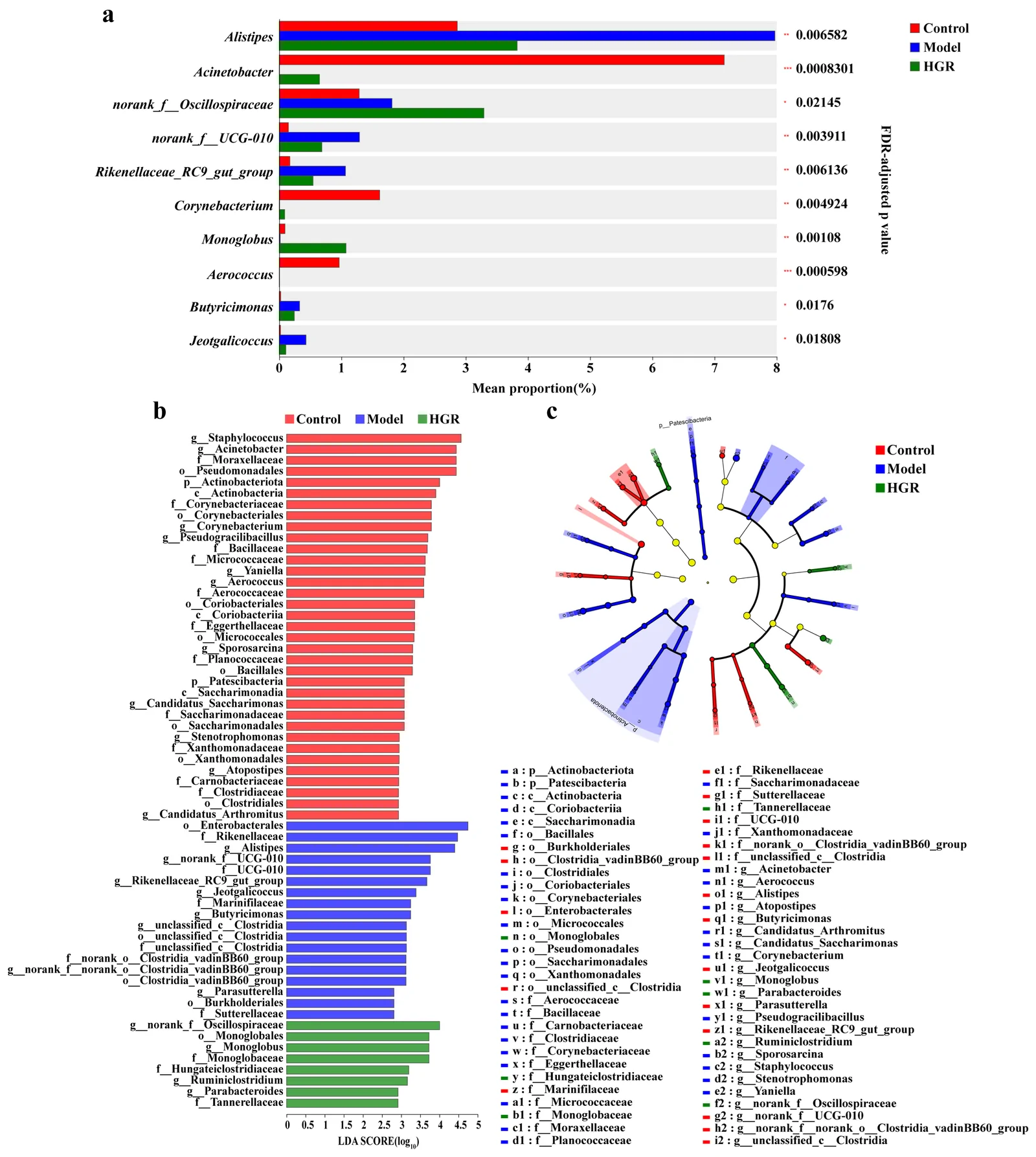
Genus-level differences and exploratory LEfSe analysis of the cecal microbiota. (**a**) Relative abundances of bacterial genera among the Control, Model, and HGR groups. Overall differences were assessed using the Kruskal–Wallis test with FDR correction, with FDR-adjusted *p* < 0.05 considered significant. * *p* < 0.05, ** *p* < 0.01, *** *p* < 0.001. (**b**) LDA score histogram and (**c**) taxonomic cladogram of candidate discriminant taxa identified by exploratory LEfSe analysis from the phylum to genus levels, *p* < 0.05, and LDA scores > 2.0. *n* = 6 mice per group.

At the genus level (Figure 6a), the Model group had higher mean relative abundances of *Alistipes*, *norank_f__UCG-010*, *Rikenellaceae_RC9_gut_group*, *Butyricimonas*, and *Jeotgalicoccus*, and lower mean relative abundance of *Acinetobacter*, *Corynebacterium*, and *Monoglobus* than the Control group. HGR showed the opposite directional pattern relative to Model for these genera, while *norank_f__Oscillospiraceae* had a higher mean relative abundance with HGR.

LEfSe was used to identify exploratory candidate discriminant taxa from phylum to genus (Figure 6b,c). Candidate genera associated with the Control group included *Acinetobacter*, *Corynebacterium*, and *Pseudogracilibacillus*, those associated with Model included *Alistipes*, *norank_f__UCG-010*, *Rikenellaceae_RC9_gut_group*, *Jeotgalicoccus*, *Butyricimonas*, and *unclassified_c__Clostridia*. Candidate genera associated with HGR included *norank_f__Oscillospiraceae*, *Monoglobus*, *Ruminiclostridium*, and *Parabacteroides*. Collectively, HGR pretreatment induced gut microbiota changes in liver injury mice.

**Figure 7:**
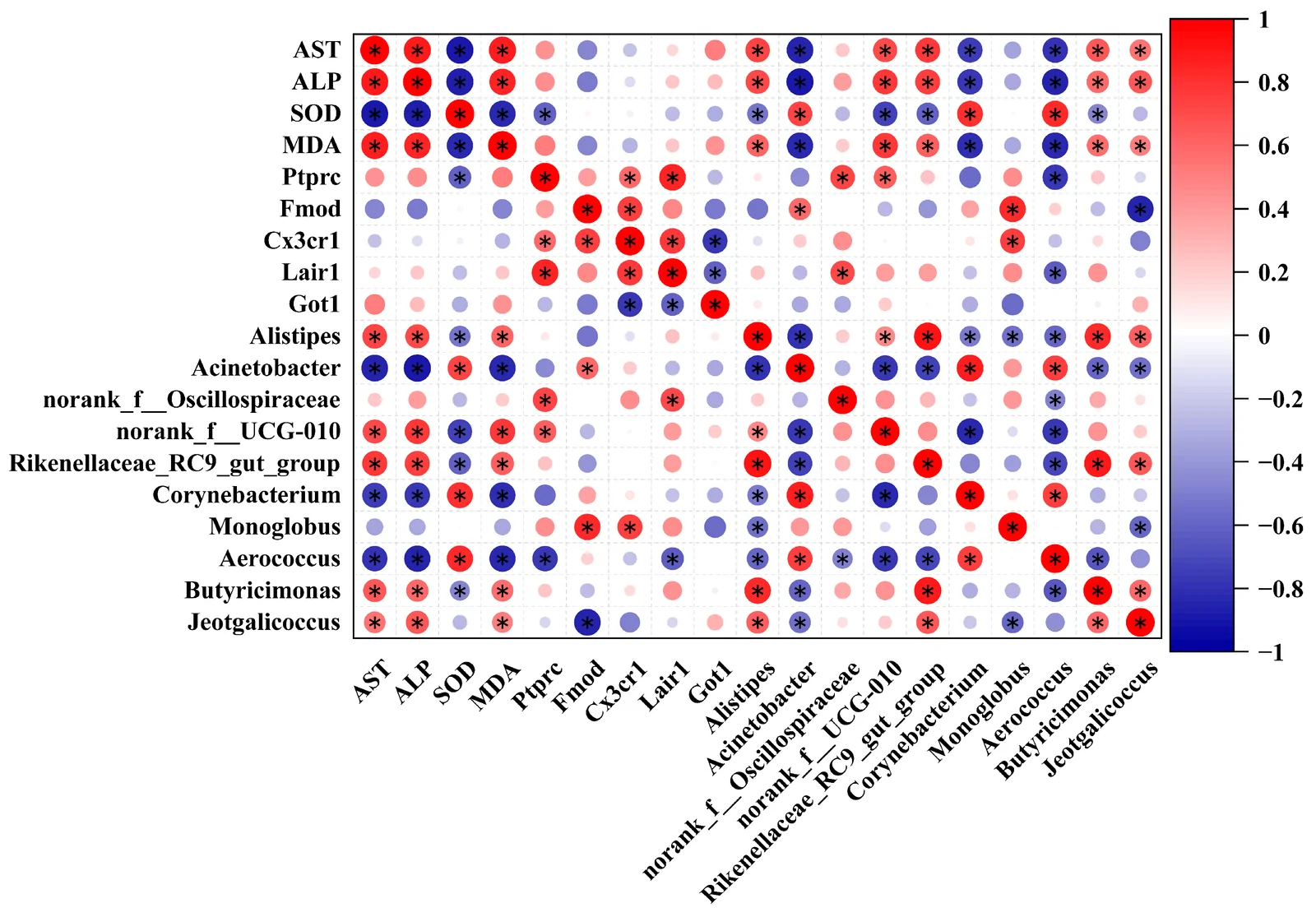
Exploratory Spearman correlations among biochemical indicators, selected hepatic transcript abundances, and bacterial genera. Individual-mouse data from Control, Model, and HGR were pooled, matched by mouse identity, and analyzed with pairwise exclusion of missing values. Biochemical data were available for 25 mice (9, 7, and 9 per group), hepatic gene-expression data for 12 mice (4 per group), and microbiota data for 18 mice (6 per group); these availability totals are not the sample size for every cross-platform correlation. Red and blue indicate positive and negative Spearman coefficients, respectively, and circle size represents the absolute coefficient. * *p* < 0.05.

Spearman correlation analysis was performed to explore the possible relationship among biochemical indicators, key DEGs, and key bacteria (Figure 7). Specifically, the serum biochemical indices (AST and ALP) exhibited positive associated with MDA levels and negative associated with SOD activity. AST and ALP were positively associated with *Alistipes*, *norank_f__UCG-010*, *Rikenellaceae_RC9_gut_group*, *Butyricimonas*, and *Jeotgalicoccus*, and negatively associated with *Acinetobacter*, *Corynebacterium*, and *Aerococcus*. Furthermore, *Acinetobacter* was positively associated with the *Fmod* transcript abundance, while *Monoglobus* was positively associated with *Fmod* and *Cx3cr1*. These nominal associations may reflect treatment-group differences.

## 4. Discussion

Fourteen days of oral GR pretreatment attenuated CCl_4_-induced acute liver injury in mice. Both doses reduced serum AST and ALP activities and increased hepatic SOD activity, while HGR also reduced the liver index and MDA content. Representative H&E images suggested less extensive necrosis and inflammatory-cell accumulation. The HGR phenotype was accompanied by hepatic gene expression and cecal microbial changes. The biochemical findings support improved hepatic oxidative status; the transcriptomic and microbial observations identify associations for further investigation.

Oxidative stress is a central event in CCl_4_ hepatotoxicity, during which reactive metabolites initiate lipid peroxidation and promote hepatocellular damage. The increased hepatic SOD activity and reduced MDA content following GR pretreatment indicate enhanced antioxidant defense and suppression of lipid peroxidation, providing a plausible explanation for the accompanying reductions in serum AST and ALP activities and histological injury. These findings are consistent with previous studies showing that peptides released during in vitro gastrointestinal digestion of germinated and heat-treated foxtail millet proteins reduced cellular reactive oxygen species and increased glutathione content and SOD activity [5]. Peptides derived from germinated, microwave-treated foxtail millet protein hydrolysates have likewise demonstrated strong antioxidant activity [23]. Recent reviews have further highlighted plant-derived protein hydrolysates, bioactive peptides, and millet biomolecules as promising food-derived modulators of hepatic oxidative and metabolic injury [24,25]. At the whole-animal level, millet bran protein hydrolysate improved metabolic liver injury in mice with non-alcoholic fatty liver disease [7]. By contrast, an earlier study found that proso millet protein protected rats against D-galactosamine-induced liver injury but not against CCl_4_-induced injury [26]. Differences in millet species, protein composition, processing conditions, dose, and injury mechanism may account for these divergent outcomes. Evidence from other food proteins further supports this biological activity: high Fischer’s ratio oligopeptides from goat whey alleviated CCl_4_-induced acute liver injury [27], while rice protein peptides and soybean meal peptides improved experimental alcohol-associated liver injury together with oxidative, metabolic, and microbial responses [28,29]. The present study extends this evidence to germinated foxtail millet protein isolate.

The hepatic transcriptional findings associate HGR pretreatment with amino acid metabolism, immune communication, and matrix-related responses. Cysteine and methionine metabolism is relevant to antioxidant defense because cysteine availability supports glutathione synthesis. Wang et al. demonstrated that increased hepatic cysteine catabolism impaired glutathione regeneration and worsened acetaminophen-induced liver injury [30]. This provides a rationale for investigating sulfur amino acid metabolism. The increases in *Cx3cr1* and *Lair1* expression suggest a possible connection between HGR pretreatment and the control of inflammation-associated oxidative injury. In a chronic CCl_4_ model, Aoyama et al. showed that CX3CR1 deficiency increased inflammatory-cell recruitment and cytokine production [31]. LAIR-1 transmits inhibitory signals that restrain immune-cell activation [32]. The increase in *Lair1* expression with HGR may therefore reflect an immune-regulatory response accompanying reduced hepatic injury. However, increased *Ptprc*, a leukocyte-associated transcript, does not establish increased inflammatory-cell numbers, as bulk liver RNA-seq cannot distinguish shifts in cell composition from changes in expression within cells. The TGF-β pathway association and *Fmod* expression change concern inflammatory and matrix responses to hepatic injury. Smad3 overexpression aggravated acute CCl_4_ injury [33], while oxidative stress increased fibromodulin production and fibromodulin promoted stellate-cell activation during chronic injury [34]. These experiments connect oxidative stress to matrix production and TGF-β signaling to inflammation.

HGR pretreatment was accompanied by changes in cecal microbial composition. The gut–liver axis provides a biological context for these findings, linking intestinal microbial communities with hepatic metabolism, immunity, and inflammatory signaling [11]. Heat-treated foxtail millet protein has previously been shown to modify gut microbial and metabolic profiles in mice with metabolic disease [35], while dietary millet protein improved metabolic syndrome together with changes in the gut microbiota, microbial metabolites, and intestinal-barrier function [8]. Notably, cereal vinegar sediment partly restored the gut microbiota in CCl_4_-treated mice, and the abundance of *Alistipes* was associated with AST, ALT, and MDA levels [36], which is consistent with the relationships observed in the present study. Although *Acinetobacter* includes opportunistic pathogens, reduced relative abundance of this genus has also been reported in mice with CCl_4_-induced acute liver injury [37], consistent with the decrease observed in our Model group. In the present study, *Acinetobacter* had a higher mean relative abundance in the HGR group than in the Model group and was inversely correlated with AST and ALP. *Butyricimonas* showed the opposite descriptive pattern, with a lower mean relative abundance in HGR than in Model and positive correlations with AST and ALP. Although *Butyricimonas* includes butyrate-producing species [38], positive associations with AST and histological injury scores have also been reported in rats with D-galactosamine-induced acute liver failure [39]. These observations indicate that butyrate-producing capacity alone does not predict the direction of an association with liver injury. Together, these findings describe genus-specific microbial changes accompanying improved hepatic oxidative status and reduced liver injury following HGR pretreatment.

Several limitations should be acknowledged. The study evaluated 14-day pretreatment in young male BALB/c mice and does not establish efficacy after injury, applicability to females, or human efficacy and safety. The absence of non-germinated millet protein and a matched standard protein control prevents attribution of protection specifically to germination or exclusion of effects from additional protein intake. A healthy GR-only group was not included, so baseline effects on organ indices, immunity, the hepatic transcriptome, and microbiota, as well as potential toxicity, remain unresolved. ALT was not measured, and AST and ALP activities indicate injury-associated changes rather than direct hepatic function. Outcome assessment and data analysis were unblinded, and histology was descriptive without quantitative scoring. Omics analyses excluded LGR, precluding assessment of dose-dependent molecular or microbial responses. RNA-seq does not resolve cell composition or cell-specific expression, and neither pathway activity nor the functional contributions of the selected genes were tested. Without microbiota-depletion or transplantation experiments, the microbial changes cannot be identified as mediators of protection. These issues require targeted validation before mechanistic or clinical conclusions can be drawn.

## 5. Conclusions

GR pretreatment attenuated CCl_4_-induced acute liver injury and improved hepatic oxidative status in young male mice. HGR-associated hepatic gene expression and cecal microbial changes provide candidate associations for mechanistic validation. These findings support further evaluation of GR for potential food applications.

## Data Availability

The original contributions presented in this study are included in the article. Further inquiries can be directed to the corresponding author. The transcriptomic and 16S rRNA gene sequencing data generated in this study are publicly available in the NCBI Sequence Read Archive (SRA) under BioProject accession numbers PRJNA1163738 and PRJNA1163529, respectively.

## Supplementary Materials

The following supporting information can be downloaded at: https://www.mdpi.com/article/10.3390/foods15213784/s1, Table S1: Endpoint-specific animal and sample availability. Table S2. Sample-level RNA-seq sequencing and quality-control statistics. Table S3. Primer sequences for RT-qPCR. Table S4. Between-group comparisons of biochemical, organ-index, and RT-qPCR outcomes.
