# Supplementary material for: Germinated Foxtail Millet Protein Attenuates CCl4-Induced Acute Liver Injury with Reduced Hepatic Oxidative Stress and Gut Microbiota Remodeling
Source: Foods. 2026 Oct 22;15(21):3784. doi: 10.3390/foods15213784 (PMC13762229; doi:10.3390/foods15213784)
Supplement: Supplementary file 1 [file foods-15-03784-s001.zip › foods-4599628-supplementary.pdf]

Germinated Foxtail Millet Protein Attenuates CCl_4-Induced Acute Liver Injury with Reduced Hepatic Oxidative Stress and Gut Microbiota Remodeling

Jingjing Li [1,3,4], Yunpeng Shen [1,3,4], Fangfang Min [1,3,4], Xiao Chen [1,3,4], Hongge Yu [1,3,4], Tian Yu [1,3,4], Zicheng Yin [1,3,4] and Sha Huang [2,*]

1 State Key Laboratory of Food Science and Resources, Nanchang University, Nanchang 330047, China.

2 Lishui Institute of Agriculture and Forestry Sciences, Lishui 323000, China

3 College of Food Science and Technology, Nanchang University, Nanchang 330031, China.

4 Sino-German Joint Research Institute, Nanchang University, Nanchang 330047, China.

* Co-corresponding author.

Sha Huang, Lishui Institute of Agriculture and Forestry Sciences, Lishui 323000, China; E-mail: huang-sha_elaine@163.com.

Foxtail millet processing and preparation of the protein isolate

Foxtail millets were washed and soaked in water for 12 h at room temperature. Then, the soaked seeds were germinated in a climate incubator (Sanfa Co., Ltd., Shanghai, China) at 30 °C for 3 days with a relative humidity of 90%. Germinated millet seeds were dried at 40 °C for 24 h to a final moisture content of 7−8%, ground in a lab grinder (Leimai Co., Ltd., Guangdong, China), and then passed through a 40-mesh sieve to produce flour.

Germinated foxtail millet protein (GR) was extracted with water (flour: water = 1: 10) at pH 11.0 using a solution 2 M NaOH and a continuous agitation speed of 500 rpm for 2 h. Flour suspension was centrifuged at 9000 × g for 30 min and the clarified supernatants were pooled and adjusted to pH 4.0 using 2 M HCl. Protein precipitate was centrifuged at 9000 × g for 30 min. phytochemicals were extracted using 1:20 (w/v) solid-to-liquid ratio using ethanol as solvent for 2 h at room temperature in the dark. Phenolic compounds were determined in alcoholic extracts by Folin-Ciocalteau method. The extraction procedure was repeated until phenolic compounds were not detected in alcoholic extracts. The mixture was filtered using filter paper. Finally, the pellet was freeze-dried and stored at -20 °C. GR denotes this freeze-dried protein isolate, which contained 80.93% protein (w/w) as estimated by the BCA assay. The same preparation batch described in the previous study [1] was used, and no enzymatic hydrolysis was performed before administration.

Correlation analysis

Exploratory Spearman correlation analyses were performed in Origin using pooled individual-mouse data from the Control, Model, and HGR groups. Biochemical indicators were available for 9, 7, and 9 mice, respectively; selected hepatic gene-expression data for 4 mice per group; and microbiota data for 6 mice per group. Measurements were matched by mouse identity, and missing values were excluded pairwise. p values were not adjusted for multiple testing; therefore, associations with $p < 0.05$ were considered nominal and exploratory.

Table S1. Endpoint-specific animal and sample availability.

Endpoint	Control	Model	LGR	HGR
Initial allocation	9	9	9	9
Terminal organ indices and biochemical measurements	9	7	7	9
Descriptive H&E assessment	3	3	3	3
Liver RNA-seq	4	4	Not analyzed	4
Cx3cr1 and *Got1* RT-qPCR	4	4	Not analyzed	4
Cecal 16S rRNA gene sequencing	6	6	Not analyzed	6

Numbers indicate individual mice. H&E used three fields per mouse at two magnifications of the same regions. Model and LGR each lost two mice before terminal sampling. Correlations exclude LGR and use mouse-matched subsets.

Table S2. Sample-level RNA-seq sequencing and quality-control statistics.

Sample	Retained reads (%)	Raw Q30 (%)	Total mapping (%)	Clean data (Gb)
Control_1	93.87	94.55	96.22	6.11
Control_2	93.81	95.20	95.94	6.59
Control_3	94.08	94.36	96.15	7.00
Control_4	93.53	95.01	94.82	6.90
Model_1	93.79	94.60	96.20	5.38
Model_2	94.11	94.64	96.46	5.34
Model_3	93.72	94.80	96.35	6.47
Model_4	94.01	94.85	96.39	6.22
HGR_1	94.00	94.92	96.88	6.54
HGR_2	93.81	95.17	96.61	6.63
HGR_3	94.00	94.80	96.51	5.09
HGR_4	93.99	94.59	96.64	5.87

Four independent liver samples were analyzed in each of the Control, Model, and HGR groups. Retained reads (%) indicates the percentage of raw reads retained after filtering. Raw Q30 (%) indicates the percentage of raw bases with a Phred quality score $\geqslant$ 30. Total mapping (%) indicates the percentage of clean reads aligned to the reference genome. Gb denotes 10^9 bases.

Table S3. Primer sequences for RT-qPCR.

Gene	Forward (5'—3')	Reverse (5'—3')
Cx3cr1	GAGTATGACGATTCTGCTGAGG	CAGACCGAACGTGAAGACGAG
Got1	TTCTTAGGGCGATGGTACAATGG	CAATAGGGCCGAATGTCCTTAAA
β-actin	GTGACGTTGACATCCGTAAAGA	GCCGGACTCATCGTACTCC

Table S4. Between-group comparisons of biochemical, organ-index, and RT-qPCR outcomes.

Outcome (unit)	Comparison	Mean difference	Tukey-adjusted 95% CI	Tukey-adjusted *p* value
Liver index (%)	Control vs Model	-0.9307	-1.210 to -0.6510	<0.0001
	Model vs LGR	0.03843	-0.2582 to 0.3351	0.9845
	Model vs HGR	0.3886	0.1089 to 0.6683	0.0038
Spleen index (%)	Control vs Model	0.01859	-0.03205 to 0.06923	0.7494
	Model vs LGR	-0.007000	-0.06071 to 0.04671	0.9842
	Model vs HGR	-0.02970	-0.08034 to 0.02094	0.3943
Serum AST (U/L)	Control vs Model	-88.38	-98.52 to -78.24	<0.0001
	Model vs LGR	23.85	13.10 to 34.61	<0.0001
	Model vs HGR	23.26	13.12 to 33.40	<0.0001
Serum ALP (U/L)	Control vs Model	-111.3	-127.8 to -94.73	<0.0001
	Model vs LGR	27.83	10.28 to 45.37	0.0009
	Model vs HGR	35.13	18.58 to 51.67	<0.0001
Cx3cr1 mRNA relative expression	Control vs Model	0.4280	0.04650 to 0.8096	0.0294
	Model vs HGR	-1.454	-1.836 to -1.072	<0.0001
Got1 mRNA relative expression	Control vs Model	-0.7432	-1.326 to -0.1605	0.0152
	Model vs HGR	0.6883	0.1056 to 1.271	0.0228
Hepatic SOD (U/mgprot)	Control vs Model	37.46	30.88 to 44.03	<0.0001
	Model vs LGR	-17.12	-24.09 to -10.14	<0.0001
	Model vs HGR	-9.826	-16.40 to -3.250	0.0018
Hepatic MDA	Control vs Model	-11.50	-13.56 to -9.448	<0.0001

(nmol/mgprot)	Model vs LGR	1.003	-1.178 to 3.184	0.5979
	Model vs HGR	3.579	1.523 to 5.635	0.0003

Only comparisons involving the Model group are shown. Mean differences equal the mean of the first-listed group minus that of the second-listed group. p values and 95% confidence intervals were adjusted using Tukey's multiple-comparisons test across all pairwise comparisons within each outcome: six comparisons among four groups for organ indices, AST, ALP, SOD, and MDA, and three comparisons among Control, Model, and HGR for *Cx3cr1* and *Got1* RT-qPCR. Organ indices, AST, ALP, SOD, and MDA included Control, n = 9; Model, n = 7; LGR, n = 7; and HGR, n = 9. RT-qPCR included n = 4 mice per group. Values returned as $p < 0.0001$ are retained as inequalities rather than treated as exact p values.

References

1. Hu, S., et al., *Antioxidant and Anti-Inflammatory Potential of Peptides Derived from In Vitro Gastrointestinal Digestion of Germinated and Heat-Treated Foxtail Millet (Setaria italica) Proteins*. Journal of Agricultural and Food Chemistry, 2020. **68**(35): p. 9415-9426.
